# Supervised physical exercise to improve the quality of life of cancer patients: the EFICANCER randomised controlled trial

**DOI:** 10.1186/s12885-015-1055-x

**Published:** 2015-02-06

**Authors:** Aintzane Sancho, Sergio Carrera, Marisol Arietaleanizbeascoa, Veronica Arce, Nere Mendizabal Gallastegui, Anna Giné March, Aitor Sanz-Guinea, Araceli Eskisabel, Ana Lopez Rodriguez, Rosa A Martín, Guillermo Lopez-Vivanco, Gonzalo Grandes

**Affiliations:** 1 Department of Oncology, Cruces University Hospital, Basque Health Service (Osakidetza), Barakaldo, Bizkaia Spain; 2 Primary Care Research Unit of Bizkaia, Basque Health Service (Osakidetza), Bilbao, Bizkaia Spain; 3 Luxana-Barakaldo Health Centre, Basque Health Service (Osakidetza), Barakaldo, Bizkaia Spain; 4 Basauri-Ariz. Health Centre, Basque Health Service (Osakidetza), Basauri, Bizkaia Spain; 5 Algorta-Bidezabal Health Centre, Basque Health Service (Osakidetza), Getxo, Bizkaia Spain

**Keywords:** Breast cancer, Colorectal cancer, Lung cancer, Metastasis, Physical exercise, Quality of life

## Abstract

**Background:**

The optimal form of exercise for individuals with cancer has yet to be identified, but there is evidence that exercise improves their quality of life. The aim of this study is to assess the efficacy and efficiency of an innovative physical exercise programme, for individuals undergoing chemotherapy for breast, gastrointestinal or non-small cell lung tumours, for improving quality of life, reducing level of fatigue, and enhancing functional capacity over time.

**Design/Methods:**

We will conduct a clinical trial in 66 patients with stage IV breast, gastrointestinal or non-small cell lung cancer, recruited by the Department of Oncology of the referral hospital from 4 primary care health centres of the Basque Health Service (Osakidetza). These patients will be randomised to one of two groups. The treatment common to both groups will be the usual care for cancer: optimized usual drug therapies and strengthening of self-care; in addition, patients in the intervention group will participate in a 2-month exercise programme, including both aerobic and strength exercises, supervised by nurses in their health centre. The principal outcome variable is health-related quality of life, measured blindly with the 30-item European Organization for the Research and Treatment of Cancer Core Quality of Life Questionnaire and Short Form-36 four times: at baseline, and 2, 6 and 12 months later. The secondary outcome variables are fatigue (Functional Assessment of Chronic Illness Therapy-Fatigue questionnaire), functional capacity (6-Minute Walk Test and cardiorespiratory test), muscle strength (hand-held dynamometry and sit-to-stand test), radiological response to treatment (Response Evaluation Criteria In Solid Tumors) and progression-free and overall survival. Age, sex, diagnosis, chemotherapy regimen, Eastern Cooperative Oncology Group performance status and smoking status will be considered as predictive variables. Data will be analysed on an intention-to-treat basis, comparing changes at each time point between groups, adjusting for baseline values by analysis of covariance.

**Discussion:**

As well as achieving the objectives set, this study will provide us with information on patient perception of the care received and an opportunity to develop a project based on collaborative action between the primary care and oncology professionals.

**Trial registration:**

ClinicalTrials.gov Identifier: NCT01786122 Registration date: 02/05/2013.

## Background

Cancer is the second most common cause of death in industrialised countries [1]. In the coming decades, it is expected to become the first cause of morbidity and mortality across the world and it already is in the Basque Country. In 2008, there were 12.7 million new cases of cancer worldwide, the most common type being lung cancer, followed by breast and colorectal cancers. It is estimated that the worldwide incidence of cancer will reach 21 million new cases by 2030 [2,3].

At the same time, great advances in the survival of cancer patients highlight the need to keep their quality of life as high as possible [4]. Hence, though for many years survival has been the most important factor in treatment selection, increasing importance is given to patient quality of life and complementary therapies are used to combine the promotion of physical wellness with meeting holistic and psychosocial needs [5-7]. Physical exercise has become the cornerstone of this approach in many diseases [8], and it is plausible that it may help mitigate some of the adverse effects of treatments in cancer: decreasing fatigue, increasing cardiorespiratory fitness and physical condition, and strengthening the immune system, and together with these, reducing recurrence rates, extending survival and improving quality of life [4,5,8-12]. Nevertheless, researchers have only recently started to study its effect in cancer patients [9].

The physical and psychosocial functioning of cancer patients is impaired by the disease itself and the toxicity of treatments [13], and in parallel their quality of life deteriorates [14,15]. They become increasingly easily fatigued, loose muscle mass and have generalised muscle weakness as well as lower exercise tolerance [13,16]. Consequently, patients enter a vicious circle of progressively increasing fatigue, dyspnoea and declining functional capacity to perform activities of daily living [17,18].

On the basis of research to date, we can hypothesise that physical exercise has a positive effect on quality of life, improves physical condition and reduces fatigue in cancer patients [11,19-23]. However, the scientific evidence available is still limited, few studies having been conducted and participants in these studies not being representative of all cancer patients. The studies have been small, sometimes without suitable control groups and, to date, have focused on the survival phase, in which palliative care is the main priority, rather than the treatment phase. Nevertheless, some studies have found that patients who exercise during the treatment phase improve their functional capacity and experience less emotional distress and fatigue [24-28]. On the other hand, it is not yet clear what would be the optimal structure for an exercise programme, in terms of type, duration, frequency and intensity, to improve quality of life in cancer patients [29-33]. It seems that the combination of aerobic exercise and strength training improves muscle function, reduces fatigue and improves quality of life during treatment [34-36]. Nevertheless, more studies are required to demonstrate that cardiovascular training combined with strength training is beneficial for patients diagnosed with cancer at all stages of the disease.

## Aim

To assess the efficacy and efficiency of an innovative physical exercise programme, for people under chemotherapy for cancer (breast, gastrointestinal and non-small cell lung cancers), for improving quality of life, reducing level of fatigue, and enhancing functional capacity, compared to usual care alone.

### Primary objectives

- To measure changes in health-related quality of life (HRQOL) between baseline and 2 months in intervention and control groups, assessing the difference in HRQOL between the groups, which is attributable to exercise.

### Secondary objectives

- To measure changes in functional capacity and level of fatigue in the intervention and control groups, assessing differences between the groups, which are attributable to exercise.

- To explore whether effects attributable to the intervention vary between subgroups of participants as a function of age, sex, cancer stage, histological findings, radiotherapy or chemotherapy regimens, as well as whether any of these variables play a role as confounders.

- To investigate whether any differences observed at 2 months are maintained in the longer term, in particular, at 6 and 12 months.

## Methods/design

### Study design

**Figure 1:**
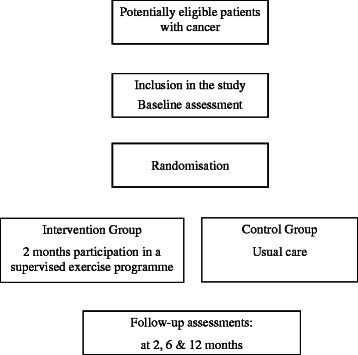
**Study flowchart.**

This is a parallel-group randomised controlled clinical trial in patients with breast, gastrointestinal or non-small cell lung cancer at stage IV of the disease. The treatment common to both groups will be the usual care for cancer, namely, optimized usual drug therapies and strengthening of self-care. The intervention group will also receive an intervention based on a supervised exercise programme led by nurses combined with education on healthy living. The patients included in the study will pass through two successive phases: a “treatment optimization phase” and a “phase for follow-up and assessment of results”. Patients are to be followed-up for 1 year, with four blind assessments: at baseline, and 2, 6 and 12 months later (Figure 1).

### Study setting

The Department of Oncology at Cruces University Hospital will recruit patients from four primary care health centres of the Basque Health Service (Osakidetza).

Each health centre has a well-established infrastructure including:

- A laboratory for measuring physical condition with cycle ergometers, treadmill ergometers, weights, dumbbells, dynamometers, pulse oximeters, electrocardiographs, anthropometric measuring instruments, blood gas analysers, and heart rate monitors, as well as defibrillators and systems for data management, among other equipment.
- Shared databases.
- Integrated data management systems based on a private virtual network connecting all the collaborating centres.

As the centre coordinating the study and provider of methodological support, the Primary Care Research Unit has:

- A license for the SAS software to perform all the statistical analyses.
- Information technology facilities, as well as premises for carrying out training activities and coordination meetings.
- Technical secretarial support.

### Study population

#### Inclusion criteria

- Age of 18–70 years old.
- Histologically confirmed diagnosis of stage IV breast, gastrointestinal or non-small cell lung cancer and an Eastern Cooperative Oncology Group (ECOG) performance status (PS) of 0 or 1.
- Usual first line chemotherapy treatment for the type of cancer in question.
- Adequate renal and liver function and blood parameters.

#### Exclusion criteria

- Brain metastases.
- High risk of fracture (bone metastasis).
- Decompensated heart disease, uncontrolled hypertension (systolic blood pressure >200 or diastolic blood pressure >110 mm Hg), heart failure (New York Heart Association Class II or greater), or constrictive pericarditis.
- Other health problems in which physical exercise is contraindicated, at the discretion of the researchers.
- Regular physical activity (150 min of moderate or 75 min of vigorous physical activity/week).

### Recruitment

For recruiting patients, the hospital’s Department of Oncology will establish a system for identifying patients discharged with stage IV breast, gastrointestinal or non-small cell lung cancers. The oncologists will tell patients about the study, invite them to participate and give them a written informed consent form, as well as informing the doctor in charge of the study in the patients’ health centre. Patients who agree to participate will be included in the study once they have signed the informed consent form and baseline data have been collected. They will be invited to an inclusion visit at which a nurse will carry out this baseline assessment and record the data.

### Randomisation

Randomization will be performed centrally, by phoning the Primary Care Research Unit, once baseline measurements have been taken at the health centres. Individuals independent of the organisation responsible for the study management and researchers will randomize patients using a computer-based random number generator. Patients will be registered and allocated on a 1:1 basis to one of the two study arms.

### Protocol for the control group

Patients in the control group will all receive the usual treatment for the type of cancer they have been diagnosed with (e.g., platinum-based chemotherapy for non-small cell lung cancer), following standard oncological criteria. They will be assessed before, during and after chemotherapy in accordance with the established protocol.

### Protocol for the intervention group

The intervention group treatment differs from that of the controls in that, in addition to the usual care, they will participate in a programme based on continuous and moderate-intensity interval aerobic exercise combined with exercises for muscle strength and joint mobility. The aerobic exercise will be performed on a cycle ergometer, this system being more suitable than treadmill exercise for cancer patients, as adverse effects of their pharmacological treatment may cause them to have difficulty with activities requiring balance and coordination. The exercise programme takes into account the principles of progression and individualization, as well precautions regarding exercise by cancer patients.

#### Phase 1 (8 weeks, 3 sessions/week)

**Figure 2:**
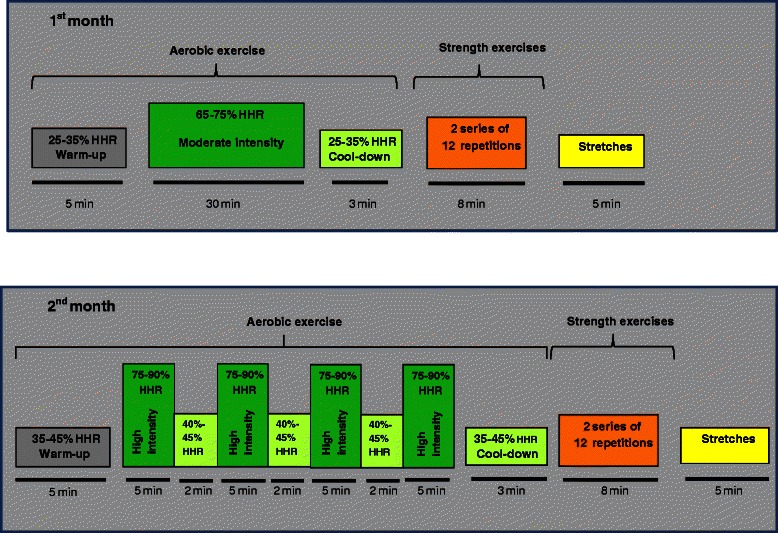
**Exercise programme of the intervention group.**

For the first 2 months, patients will follow a progressive exercise programme consisting of 24 sessions, the intensity being lower at the beginning and increasing in the second month (Figure 2). Two of the sessions each week will take place under the supervision of the nurse in the laboratory, while the third session is to be carried out independently in the area around the health centre, with the aim of promoting patient independence, and for this, patients are to wear a heart rate monitor pre-programmed by the nurse. Each laboratory session will include health education, as well as aerobic exercise and muscle strength exercises.

#### Education on healthy living

Patients will receive advice on the type of exercise to do independently and be taught how to measure their heart rate and to use the heart rate monitor with heart rate alerts.

#### Aerobic exercise

In each session, the intensity of the exercise is adapted to the physical condition of patient. The activity will be monitored using the Borg scale to rate the patient’s perceived exertion before and after each session and by watching for the appearance of symptoms. During the first month, patients will perform continuous aerobic exercise at a constant intensity equivalent to 60-70% of their heart rate reserve (HRR). In the second month, the intensity of the exercise will be increased, patients performing aerobic interval exercise, alternating 5-minute periods of very high intensity at 75-90% of their HRR with 2-minute periods of active resting at 40% of their HRR. This aerobic exercise is to be carried out before strength exercises to ensure cardiovascular and muscular warm-up.

#### Strength and joint mobility exercises

In each session, patients will carry out 8 exercises each with 2 series of 8–12 repetitions. In this way, the muscles involved in activities of daily living will be exercised (biceps, triceps, deltoid and trapezius muscles; knee and hip extensors and flexors; and deep abdominal and back muscles related to posture), applying a different weight for each muscle group. Dumbbells and exercise resistance bands will be used for upper limbs and lower limbs, respectively.

At the end of the supervised exercise, to assess the acceptance of this type of intervention, the nurse will interview patients about their experience of the programme. This interview will be audio recorded and later analysed by a sociologist experienced in qualitative analysis.

#### Phase II

It is envisaged that the training received in Phase I will help patients to maintain an exercise routine. They will be trained to carry out an exercise programme similar to that of the intervention, independently and indefinitely, using community facilities.

### Outcome measures

#### Primary outcome measure

Changes in HRQOL after 2 months to assess the results of Phase I. HRQOL will be measured blindly using the 30-item European Organization for the Research and Treatment of Cancer Core Quality of Life Questionnaire (EORTC QLQ-C30). This questionnaire is specific for assessing HRQOL of patients with cancer, asking how they have been feeling in the previous 7 days. The 30 items cover 9 multi-item scales: 5 functional scales (physical, role, cognitive, emotional and social functioning) and 3 symptom scales (fatigue, pain, and nausea/vomiting), as well as a global health status and quality of life scale. It also contains ratings of certain symptoms (dyspnoea, insomnia, appetite loss, constipation and diarrhoea). The raw scores are linearly transformed to standard scores between 0 and 100. This questionnaire has been considered valid and reliable in multiple studies on cancer patients. In addition, we will use the Short Form-36 (SF-36) generic quality of life questionnaire. Both questionnaires have been validated for the Spanish population [37,38].

#### Secondary outcome measures

- Degree of fatigue measured using the Functional Assessment of Chronic Illness Therapy-Fatigue questionnaire (FACIT-Fatigue).
- Radiological response to treatment (Response Evaluation Criteria In Solid Tumors, RECIST) [39].
- Functional capacity (6-Minute Walk Test; cardiorespiratory fitness test measured using a cycle ergometer submaximal exercise test).
- Muscle strength (hand-held dynamometry and 5-times sit-to-stand test).
- Progression-free and overall survival.

##### Costs

The costs will be assessed from the perspective of the programme. That is, we will only include healthcare costs related to the intervention, in line with the recommendations of the National Institute for Health and Clinical Excellence (NICE) in the UK. We will use a bottom-up approach to estimate costs. This methodology consists of recording the resources used by each centre and converting these into currency units. The costs will be classified as: 1) time dedicated to the programme by healthcare staff involved, namely, nurses; 2) consumables; and 3) structural costs.

Predictive (or confounding) variables.

Age, sex, type of cancer (breast, gastrointestinal or lung), chemotherapy regimen, ECOG PS, and smoking status.

### Adverse events

An external committee will review and compare all non-serious adverse events, while researchers will be obliged to report any serious adverse events to the research unit by fax. A coordination committee with access to all the information it needs will undertake preliminary analyses of the data to monitor the safety of the programme. This committee will be composed of individuals that are independent of the organisation responsible for the study management and members of the research team including the study coordinator, all blind to patient allocation. In addition to serving on the committee, the coordinator will phone the participating health centres daily, to check on the progress of the study, report weekly on this progress to the principal investigator of the study, produce a monthly report with the study data, and make recommendations to the study management team.

### Follow-up period

One year from the beginning of the intervention.

### Sample size

We estimate that we need to analyse 26 patients per group to detect a difference of 20 points in the global scales and subscales of the EORTC QLQ-C30 as significant, a difference considered to be important from the patient point of view (level of significance, 0.05; power, 0.80 and standard deviation, 25). For this reason, we will include 33 patients per group, that is, a total of 66, allowing for a loss to follow-up of 20%. Given that in the pilot 66% of candidates signed the informed consent form, we need to invite a total of 100 patients to participate.

### Statistical analysis

The primary outcome variable has been found to have a normal distribution in previous studies; for variables that are non-normally distributed, non-parametric tests will be used and models built suitable for their type of distribution. Analyses will be conducted on an intention-to-treat basis, and changes between groups compared at each time point of the follow-up, by analysis of covariance adjusting for baseline levels. The effect attributable to the intervention will be estimated by assessing the differences in improvement in the groups, with 95% confidence intervals. Stratified analysis and statistical models, linear for continuous outcome variables and logistic for dichotomous variables (quantitative categorized variables), will be used to adjust these comparisons for potential confounding factors. To assess the change overall over the 1-year follow-up, we will estimate the effect of time on three repeated measures in each subject, using mixed linear regression models, fixed effect models (time, intervention, interaction between time and intervention) and random effect models (specific effect of each subject and each centre on the baseline and on the effect of time). These models will take into account the longitudinal nature of the data from three repeated measurements in each patient and will be extended to adjust for the putative predictive (or confounding) variables.

Estimates will be made of the incremental cost-effectiveness and cost-utility ratios, dividing the increase in cost between the groups by the increases in effectiveness and utility, respectively. Confidence intervals will be calculated for these ratios using random sampling techniques (bootstrapping). Sensitivity analysis will be performed, changing the assumptions of the analysis. All analyses will be carried out using the SAS statistical package.

### Quality control

To ensure the quality of the study data and maximise the validity and reliability of the programme and measurement of the variables, we will undertake the following:

- Produce documents for the study: operational manuals for fieldwork, and forms for registering measurements and details of the intervention.
- Store all documentation (informed consent forms, documents containing results, etc.) in locked cabinets or on a secure server.
- Provide training for those responsible for the standardisation of the study process, including specific training for nurses involved in the study, in particular, for administration of the quality of life questionnaires.
- Hold regular meetings.
- Establish a coordinating committee, as noted above, the coordinator contacting the health centres daily, requesting the information regarding the study process, and reporting to the principal investigator weekly.
- Produce progress reports monthly.

### Ethical and legal aspects

This study protocol complies with the Declaration of Helsinki and its revisions, as well as with good clinical practice. The Ethics Committee of the Basque Country approved the study in the four health centres ensuring it would be implemented in compliance with the established regulations. Regarding data confidentiality, only the study researchers have access to the data of individuals who agree to participate in the study, in compliance with the Organic Act 15/1999 of December 13, on the protection of personal data and its 2011 revision.

### Limitations

The study will have limited statistical power to detect changes of less than 10 points on the EORTC QLQ-C30 global scale and subscales, which could still be considered relevant from the patient point of view. Initially, patients were going to be recruited from 5 centres, and it was estimated that a sample of 100 per group would have been required for detecting this type difference as significant. However, given that only the coordinating centre has received funding for this study and a single centre could not take on the recruitment of that many patients, we have had to accept a smaller target sample size and recalculate the statistical power of the sample.

Further, this study does not consider mortality or hospitalisation as primary outcome measures, rather it focuses on functional capacity and quality of life as the key factors to attempt to improve in these patients.

The structure of the study means that it is not possible to adopt a double-blind design (researchers and participants); however, there will be external assessment that is independent of the data collection process.

Given that monitoring of the intervention and data collection are complex, a system for quality control will be established to ensure standardisation of the process. Further, in order to avoid the contamination of the control group, researchers in charge of data collection will be specifically trained and we will undertake a pilot study.

## Discussion

This study seeks to make an important contribution to our understanding of the therapeutic effects of exercise in cancer patients, with the goal of helping them to maintain a better quality of life. We undertake this work at a time when clinical interest in the role of physical exercise in cancer is progressively increasing and in view of the remaining gaps in our knowledge more than two decades after the first research studies in this field [40]. It will provide scientific evidence on the effect of exercise during chemotherapy, a stage that has been considered in few studies, in patients with breast gastrointestinal, or non-small cell lung tumours, most research to date having focused only on breast or colon cancer.

The study assesses a programme that could be considered ideal, in the light of current knowledge: it combines aerobic and strength exercises and is supervised by nurses to adapt the intensity of the exercise to the current functional situation of each patient, thereby taking best advantage of each session while safeguarding patient safety. At the same time, it empowers patients to self-manage their own physical training, in accordance with a self-evaluation of their functional status, their changing needs over the course of their chemotherapy and the progress of their tumour. Further, taking a social and ecological approach including the use of community resources, the programme encourages ongoing adherence to exercise by patients.

The intervention studied is a single programme for various different types of cancer that adapts to the requirements of each patient. Previously, specific physical exercise interventions have been designed for different types of cancer; however, in an editorial published in the British Medical Journal in 2011, NH Williams suggested that this approach should be changed [8]. Multiple recently published research studies and protocols propose a common intervention for different types of cancer, personalized to patient needs, which undoubtedly makes these interventions more cost-effective than those focused specifically on single types of cancer [22,25,28].

Given that one of the reasons patients refuse to participate in exercise programmes is a need to travel [41], this study is based on “low-tech” facilities, easily accessible to patients in their own primary care health centres. To maintain the effect of exercise in the long term, it is essential to ensure adherence to the programme.

Regardless of whether the exercise turns out to be effective, we can be confident that the programme is safe and does not represent any additional risk for cancer patients. Specifically, other similar studies have reported no adverse effects associated with exercise [32,42,43].

This study offers the possibility of assessing the experience of patients with an exercise programme and demonstrates well-coordinated work of the oncology and primary care units in tertiary prevention. Indeed, given our positive experience, we are already considering developing this type of physical exercise programme for individuals with other chronic disorders.

In summary, our hypothesis is that a programme of physical exercise coordinated between the oncology and primary care units, supervised at health centres by nurses to safeguard patient safety but which can be can be continued in the community setting, and common for all types of cancer while tailored to meet individual patient needs, will be effective in improving the quality of life of patients with cancer as well as being cost-effective.

## References

[1] World Health Organization (2008). The Global Burden of Disease: 2004 Update.

[2] Ferlay J, Shin H-R, Bray F, Forman D, Mathers C, Parkin DM (2010). Estimates of Worldwide Burden of Cancer in 2008: GLOBOCAN 2008. Int J Cancer.

[3] Jemal A, Bray F, Center MM, Jacques F, Ward E, Forman D (2011). Global Cancer Statistics. CA Cancer J Clin.

[4] Coleman MP, Forman D, Bryant H, Butler J, Ratchet B, Marine C (2011). Cancer survival in Australia, Canada, Denmark, Norway, Sweden, and the UK, 1995–2007 (the International Cancer Benchmarking Partnership): an analysis of population-based cancer registry data. Lancet.

[5] Christiane P, Schulz T, Michna H (2002). Exercise in Cancer Therapy. Eur J Sport Sci.

[6] Deng GE, Rausch SM, Jones LW, A G, Kumar NB, Greenlee H (2013). Complementary Therapies and Integrative Medicine in Lung Cancer: Diagnosis and Management of Lung Cancer, 3rd Ed: American College of Chest Physicians Evidence-based Clinical Practice Guidelines. Chest J.

[7] Rueda JR, Solà I, Pascual A, Subirana Casacuberta M (2011). Non-invasive interventions for improving well-being and quality of life in patients with lung cancer. Cochrane Database Syst Rev.

[8] Williams NH (2011). Promoting Physical Activity in Primary Care. BMJ.

[9] Jones LW, Peppercorn J (2010). Exercise Research: Early Promise Warrants Further Investment. Lancet Oncol.

[10] Lakoski SG, Eves ND, Douglas PS, Jones LW (2012). Exercise Rehabilitation in Patients with Cancer. Nat Rev Clin Oncol.

[11] Cramp F, Byron-Daniel J (2012). Exercise for the management of cancer-related fatigue in adults. Cochrane Database Syst Rev.

[12] Galvão DA, Newton RU (2005). Review of Exercise Intervention Studies in Cancer Patients. J Clin Onco.

[13] Gilliam Laura AA, St Clair DK (2011). Chemotherapy-induced Weakness and Fatigue in Skeletal Muscle: The Role of Oxidative Stress. Antioxid Redox Signal.

[14] Herman JM, Narang AK, Griffith KA, Zalupski MM, Reese JB, Gearhart SL (2013). The Quality-of-life Effects of Neoadjuvant Chemoradiation in Locally Advanced Rectal Cancer. Int J Radiat Oncol Biol Phys.

[15] Fialka-Moser V, Crevenna R, Korpan M, Quittan M (2003). Cancer Rehabilitation: Particularly with Aspects on Physical Impairments. J Rehabil Med.

[16] Del Fabbro E, Dalal S, Bruera E (2006). Symptom Control in Palliative care–Part II: Cachexia/anorexia and Fatigue. J Palliat Med.

[17] Curt GA (2000). Impact of Fatigue on Quality of Life in Oncology Patients. Semin Hematol.

[18] Lucía A, Earnest C, Pérez M (2003). Cancer–related Fatigue: Can Exercise Physiology Assist Oncologists?. Lancet Oncol.

[19] Mishra SI, Scherer RW, Snyder C, Geigle PM, Berlanstein DR, Topaloglu O (2012). Exercise Interventions on Health-related Quality of Life for People with Cancer During Active Treatment. Cochrane Database Syst Rev.

[20] Puetz TW, Herring MP (2012). Differential Effects of Exercise on Cancer-Related Fatigue During and Following Treatment: A Meta-Analysis. Am J Prev Med.

[21] Fong DYT, Ho JWC, Hui BPH, Lee AM, Macfarlane DJ, Leung SSK (2012). Physical Activity for Cancer Survivors: Meta-analysis of Randomised Controlled Trials. BMJ.

[22] Velthuis MJ, Agasi-Idenburg SC, Aufdemkampe G, Wittink HM (2010). The Effect of Physical Exercise on Cancer-related Fatigue During Cancer Treatment: a Meta-analysis of Randomised Controlled Trials. Clin Oncol.

[23] 23- Tomlinson D, Diorio C, Beyene J, Sung L. Effect of Exercise on Cancer-Related Fatigue: A Meta-analysis. American Journal of Physic and Medicine Rehabilitation 2014, 16 [Epub ahead of print] 10.1097/PHM.0000000000000083 24743466

[24] Andersen C, Rørth M, Ejlertsen B, Stage M, Møller T, Midtgaard J (2013). The Effects of a Six-week Supervised Multimodal Exercise Intervention During Chemotherapy on Cancer-related Fatigue. Eur J Oncol Nurs.

[25] Adamsen L, Quist Q, Andersen C, Møller T, Herrstedt J, Kronborg D (2009). Effect of a Multimodal High Intensity Exercise Intervention in Cancer Patients Undergoing Chemotherapy: Randomised Controlled Trial. BMJ.

[26] Wenzel JA, Griffith KA, Shang JJ, Thompson CB, Hedlin H, Stewart KJ (2013). Impact of a Home-Based Walking Intervention on Outcomes of Sleep Quality, Emotional Distress, and Fatigue in Patients Undergoing Treatment for Solid Tumors. Oncologist.

[27] Griffith K, Wenzel J, Shang JJ, Thompson C, Stewart K, Mock V (2009). Impact of a Walking Intervention on Cardiorespiratory Fitness, Self-reported Physical Function, and Pain in Patients Undergoing Treatment for Solid Tumors. Cancer.

[28] Van Waart H, Stuiver MM, van Harten WH, Sonke GS, Aaronson NK (2010). Design of the Physical Exercise During Adjuvant Chemotherapy Effectiveness Study (PACES): a Randomized Controlled Trial to Evaluate Effectiveness and Cost-effectiveness of Physical Exercise in Improving Physical Fitness and Reducing Fatigue. BMC Cancer.

[29] Stevinson C, Lawlor DA, Fox KR (2004). Exercise Interventions for Cancer Patients: Systematic Review of Controlled Trials. Cancer Causes Control.

[30] Markes M, Brockow T, Resch KL (2006). Exercise for Women Receiving Adjuvant Therapy for Breast Cancer. Cochrane Database Syst Rev.

[31] Riesenberg H, Lübbe AS (2009). In-patient Rehabilitation of Lung Cancer Patients--a Prospective Study. Support Care Cancer.

[32] Schmitz KH, Courneya KS, Matthews C, Demark-Wahnefried W, Galvão DA, Pinto BM (2010). American College of Sports Medicine Roundtable on Exercise Guidelines for Cancer Survivors. Med Sci Sports Exerc.

[33] Conn VS, Hafdahl AR, Porock DC, McDaniel R, Nielsen PJ (2006). A Meta-analysis of Exercise Interventions Among People Treated for Cancer. Support Care Cancer.

[34] Strasser B, Steindorf K, Wiskemann J, Ulrich CM (2013). Impact of Resistance Training in Cancer Survivors: a Meta-analysis. Med Sci Sports Exerc.

[35] Mustian KM, Peppone L, Darling TV, Palesh O, Heckler CE, Morrow GR (2009). A 4-Week Home-Based Aerobic and Resistance Exercise Program During Radiation Therapy: A Pilot Randomized Clinical Trial. J Support Oncol.

[36] De Backer IC, Van Breda E, Vreugdenhil A, Nijziel MR, Kester AD, Schep G (2007). High-intensity Strength Training Improves Quality of Life in Cancer Survivors. Acta Oncol.

[37] Alonso J, Prieto L, Anto JM (1995). La versión española del SF-36 Health Survey (Cuestionario de Salud SF-36): un instrumento para la medida de los resultados clínicos. Med Clin.

[38] Arrarás JI, Arias F, Tejedor M, Pruja E, Marcos M, Martínez E (2002). QLQ-C30 (VERSION 3.0) Quality of Life Questionnaire: Validation Study for Spain with Head and Neck Cancer Patients. Psychooncology.

[39] Eisenhauer EA, Therasse P, Bogaerts J, Schwartz LH, Sargent D, Ford R (2009). New Response Evaluation Criteria in Solid Tumours: Revised RECIST Guideline (version 1.1). Eur J Cancer.

[40] Jones LW, Alfano CM (2013). Exercise-oncology Research: Past, Present, and Future. Acta Oncol.

[41] Maddocks M, Mockett S, Wilcock A (2009). Is Exercise an Acceptable and Practical Therapy for People with or Cured of Cancer?. Cancer Treat Rev.

[42] Quist M, Rørth M, Langer S, Jones LW, Laursen JH, Pappot H (2012). Safety and Feasibility of a Combined Exercise Intervention for Inoperable Lung Cancer Patients Undergoing Chemotherapy: a Pilot Study. Lung Cancer.

[43] Albrecht TA, Taylor AG (2012). Physical Activity in Patients with Advanced-stage Cancer: a Systematic Review of the Literature. Clin J Oncol Nurs.

